# Activating Nrf-2 Signaling Depresses Unilateral Ureteral Obstruction-Evoked Mitochondrial Stress-Related Autophagy, Apoptosis and Pyroptosis in Kidney

**DOI:** 10.1371/journal.pone.0047299

**Published:** 2012-10-10

**Authors:** Shue Dong Chung, Ting Yu Lai, Chiang Ting Chien, Hong Jen Yu

**Affiliations:** 1 Department of Urology, Far East Memory Hospital, New Taipei City, Taiwan; 2 Graduate Institution of Clinical Medicine, National Taiwan University College of Medicine, Taipei, Taiwan; 3 Department of Urology, National Taiwan University Hospital and National Taiwan University College of Medicine, Taipei, Taiwan; 4 Department of Medical Research, National Taiwan University Hospital, Taipei, Taiwan; 5 Department of Life Science, National Taiwan Normal University, Taipei, Taiwan; Pennington Biomedical Research Center, United States of America

## Abstract

Exacerbated oxidative stress and inflammation may induce three types of programmed cell death, autophagy, apoptosis and pyroptosis in unilateral ureteral obstruction (UUO) kidney. Sulforaphane activating NF-E2-related nuclear factor erythroid-2 (Nrf-2) signaling may ameliorate UUO-induced renal damage. UUO was induced in the left kidney of female Wistar rats. The level of renal blood flow, cortical and medullary oxygen tension and reactive oxygen species (ROS) was evaluated. Fibrosis, ED-1 (macrophage/monocyte) infiltration, oxidative stress, autophagy, apoptosis and pyroptosis were evaluated by immunohistochemistry and Western blot in UUO kidneys. Effects of sulforaphane, an Nrf-2 activator, on Nrf-2- and mitochondrial stress-related proteins and renal injury were examined. UUO decreased renal blood flow and oxygen tension and increased renal ROS, 3-nitrotyrosine stain, ED-1 infiltration and fibrosis. Enhanced renal tubular Beclin-1 expression started at 4 h UUO and further enhanced at 3d UUO, whereas increased Atg-5-Atg12 and LC3-II expression were found at 3d UUO. Increased renal Bax/Bcl-2 ratio, caspase 3 and PARP fragments, apoptosis formation associated with increased caspase 1 and IL-1β expression for pyroptosis formation were started from 3d UUO. UUO reduced nuclear Nrf-2 translocation, increased cytosolic and inhibitory Nrf-2 expression, increased cytosolic Bax translocation to mitochondrial and enhanced mitochondrial Cytochrome c release into cytosol of the UUO kidneys. Sulforaphane significantly increased nuclear Nrf-2 translocation and decreased mitochondrial Bax translocation and Cytochrome c release into cytosol resulting in decreased renal injury. In conclusion, sulforaphane via activating Nrf-2 signaling preserved mitochondrial function and suppressed UUO-induced renal oxidative stress, inflammation, fibrosis, autophagy, apoptosis and pyroptosis.

## Introduction

Obstruction of the upper urinary tract has deleterious effects on the kidney. The histological derangements associated with obstruction are localized primarily in the tubulointerstitial areas of the kidney and include massive tubular dilation, apoptotic tubular cell deletion, and progressive tubulointerstitial fibrosis [1], [2]. Unilateral ureteral obstruction (UUO), a well-characterized hydronephrosis model, demonstrates a depressed renal blood flow in the obstructed kidney [2]. The renal tubulointerstitium demanding high oxygen consumption for active solutes transport and reabsorption is easily susceptible to oxidative stress. Increased toxic reactive oxygen species (ROS) accumulation leading to tubulointerstitial injury is frequently recognized in various kinds of kidney diseases [3], [4]. The excess ROS evoke abnormal signal transduction, cellular dysfunction, inflammatory monocyte/macrophage (ED-1) infiltration and cell death cascade in the damaged kidneys [2], [3], [5].

Increased ROS generation in the mitochondria or other intracellular compartments may induce three types of programmed cell death, apoptosis, autophagy or pyroptosis, via the execution by caspases, lysosomal proteases, or endonucleases [2], [6]–[9]. The increased ROS like O_2_^−^. and H_2_O_2_ derived from damaged mitochondria trigger apoptosis by releasing mitochondrial cytochrome C into the cytosol to increase caspase 3 activity and PARP cleavage and/or enhance autophagy by activating Beclin-1, LC3-II and ATG5-ATG12 proteins expression [6], [10]–[12]. On the other hand, pyroptosis, another type of programmed cell death distinct from apoptosis, can be triggered by increased oxidative stress or inflammation to activate inflammasome-caspase 1-IL-1β/IL-18 signaling, which subsequently results in tissue injury [8], [13], [14]. Increased caspase 1 activity and IL-1β/IL-18 secretion have been implicated in the animal models or human forms of chronic kidney diseases [14]. In this study, we suggest that increased ROS and inflammation may induce three types of programmed cell death in the UUO kidney.

Bcl-2 family members like Bax and Bcl-2 may involve apoptosis, autophagy and pyroptosis [9]. For example, cytosolic Bax translocation to mitochondria triggers cytosolic Cytochrome c release for inducing apoptosis, whereas Bcl-2 and/or Bcl-xL can block cytosolic Cytochrome c release for depressing apoptosis [15]–[17]. Bcl-2 augmentation protects renal tubular epithelial cells from oxidative injury by suppressing both autophagy and apoptosis formation and tubulointerstitial injury [18]. Bcl-2 and Bcl-xL bind and suppress NLR-family protein NALP1 for reducing caspase-1 activation and IL-1β production [19]. The inhibitor of Nrf-2 (I-Nrf-2) or Kelch-like ECH-associated protein 1 (Keap1)/NF-E2-related factor (Nrf-2) can be a sensor for oxidative stress. I-Nrf-2 or Keap1 functions as a substrate adaptor protein for degrade Nrf-2 [20], [21]. I-Nrf-2 targets anti-apoptotic Bcl-2 protein for degradation and controls cellular apoptosis [22]. Antioxidant treatment destabilizes Nrf-2-I-Nrf-2-Bcl-xL complex in mitochondria, leading to the release of Nrf-2 and increased Bcl-xL heterodimerization with Bax and reduced cellular apoptosis [23]. Nrf-2 and I-Nrf-2 also act as redox sensors to regulate autophagy formation [24], [25]. Cells via Nrf-2 regulate antioxidant response element-mediated expression of detoxifying and antioxidant enzymes and provide antioxidant and anti-inflammatory protection [26]. Activating Nrf-2 signaling provides cardioprotection, renoprotection and anti-inflammation; however, downregulating or knockout Nrf-2 abrogates such protection [13], [26]–[27]. We hypothesize that diminished nuclear Nrf-2 translocation may contribute to increased oxidative stress and mitochondrial dysfunction through Bcl-2-related mechanism in the damaged cells of UUO kidney. We also hypothesize UUO-induced inflammation and oxidative stress may enhance three types of programmed cell death, autophagy, apoptosis or pyroptosis formation, in the damaged kidney through the mitochondrial dysfunction and inhibition of nuclear Nrf-2 translocation. We further suggest that enhancements in nuclear Nrf-2 translocation and prevention in mitochondrial Cytochrome c release to cytosol may attenuate ROS-triggered autophagy, apoptosis and pyroptosis in the UUO kidney.

## Materials and Methods

### Animals

Female Wistar rats (200–250 g) were purchased from BioLASCO Taiwan Co. Ltd. (Taipei) and housed at the Experimental Animal Center, National Taiwan University, at a constant temperature and with a consistent light cycle (light from 07∶00 to 18∶00 o’clock). Food and water were provided ad libitum. All surgical and experimental procedures were approved by the Institutional Animal Care and Use Committee of National Taiwan University College of Medicine and College of Public Health and were performed in accordance with the guidelines of the National Science Council of Republic of China (NSC 1997). All efforts were made to minimize both animal suffering and the number of animals used throughout the experiment.

### Induction of Unilateral Ureteral Obstruction (UUO)

The animals were divided into three groups: sham control (n = 10), UUO group (n = 80) and UUO treated with daily i.p. sulforaphane (*R,S*-Sulforaphane, SR, LKT Laboratories, St. Paul, MN, n = 10) at the dosage of 0.7 mmoL/kg dissolved in corn oil. The selected dose was based on a chemoprotective activity in the rat subjected to hepatoxicity [28]. We have also used another antioxidant, decaffeinated green tea extract (catechins, 25 mg/kg i.p.), treatment in some rats (n = 5) for evaluating its effect on UUO-induced renal dysfunction. The catechins were purchased from Numen Biotech Co., Ltd., (Taipei, Taiwan) and catechins were composed of various types of catechins (328 mg/g of epigallocatechin gallate, 152 mg/g of epicatechin gallate, 148 mg/g of gallocatechin gallate, 132 mg/g of epicatechin, 108 mg/g of epigallocatechin, 104 mg/g of galloctechin, and 44 mg/g of catechin), which is analyzed by high performance liquid chromatography. Under avertin anesthesia (200 mg/kg body weight, i.p.), the left ureter was ligated with 4-0 silk at two locations and cut between the ligatures to prevent retrograde urinary tract infection [2]. The ligation for induction of UUO was maintained throughout the experiment at 0 h (0 hUUO), 4 h (4 hUUO), 8 h (8 hUUO), 12 h (12 hUUO), 1 d (1dUUO), 3 d (3dUUO), 5 d (5dUUO) or 7 d (7dUUO) (n = 10 in each group). A sham operation was performed in the control rats (n = 10), which had their ureters manipulated but not ligated. Four h to 7 d after the sham operation, rats were killed to obtain control kidneys.

### Measurement of Renal Blood Flow and Tissue Oxygen Tension

At the indicated time for experiments, the rats were anesthetized with urethane (1.2 g/kg, i.p.). PE-50 catheters were placed in the left carotid artery for measurements of arterial blood pressure by an ADI system (PowerLab/16S, ADI Instruments, Pty Ltd, Castle Hill, Australia) with a transducer (P23 1D, Gould-Statham, Quincy), and in the left femoral vein for administration of anesthetics when needed. Renal arterial blood flow was measured using a ultrasound flow probe (0.5VB334, Transonic System Inc., NY) placed around the left renal artery, recorded with a T206 recording system (Transonic System Inc.), and displayed on the ADI system. Cortical and medullary tissue oxygenation was simultaneously evaluated by using two oxygen sensing probes (OxyLite 2000E, Oxford Optronix Limited, Oxford, UK). In brief, initial small incisions were made in the renal capsule with a 25-G needle to allow easy passage of two 230-µm-diameter fiber-optic probes (Oxford Optronix) into the parenchyma for monitoring of tissue O_2_ tension. To minimize possible hematoma formation at the determined regions, the probes were inserted to the required depths (0.5, and 3.5 mm) with stereotaxic micromanipulators. These probes were placed perpendicular to the kidney surface and these depths were selected due to them anatomically corresponding to the cortex and outer medulla as described previously [29]. This consistent placement ensured that the same region was always being measured in different animals. The probes were connected to OxyLite monitors to record cortical and medullary O_2_ tension at 1-s intervals. To ensure model stability, the animals were monitored for 30 min after instrumentation and before the experimental intervention. All physiological data were collected using a 16-Channel http://ajprenal.physiology.org/cgi/redirect-inline?ad=PowerlabPowerlab 16PC system (http://ajprenal.physiology.org/cgi/redirect-inline?ad=AD%20InstrumentsAD Instruments).

At the end of each experiment, the rats were sacrificed with an overdose of KCl. The kidneys were removed and divided into two parts. One part was stored in 10% neutral buffered formalin for pathologic and immunohistochemic assay, and the other was freshly prepared for nuclear, mitochondrial or cytosolic protein isolation and stored at -80°C for further assay.

### *In vivo* ROS Recording

The *in vivo* ROS measurement was detected from the kidney by intravenous infusion of 2-Methyl-6-(4-methoxyphenyl)-3,7-dihydroimidazo-[1,2-a]-pyrazin-3-one- hydrochloride (MCLA) (0.2 mg/ml/h, TCI-Ace, Tokyo Kasei Kogyo Co. Ltd., Tokyo, Japan) and examined by a Chemiluminescence Analyzing System (CLD-110, Tohoku Electronic In. Co., Sendai, Japan) [3].

### *In situ* Detection of Oxidative Stress, Inflammation, Autophagy, Apoptosis and Pyroptosis

Increased oxidative stress might be associated with the inflammation, production of apoptosis, autophagy and pyroptosis. We evaluated 3-nitrotyrosine (3-NT) to localize oxidative stress production, ED-1 stain for inflammation, Beclin-1 stain for autophagy, terminal deoxynucleotidyl transferase-mediated nick-end labeling (TUNEL) for apoptosis and caspase/IL-1β stains for pyroptosis in the paraffin-embedded sections of kidney tissues. Renal sections obtained from 10% formalin fixation and paraffin embedding were deparaffinized, rehydrated, and stained immunohistochemically for 3-NT by incubation with a polyclonal antibody (Alpha Diagnostic International; San Antonio, TX, USA) diluted at 1∶50. The value of brown deposits/total section area in the 3-NT was counted by Adobe Photoshop 7.0.1 image software analysis.

For ED-1 staining, the tissue sections were incubated overnight at 4°C with a mouse anti-rat antibody to ED-1 (BioSource Intermational,Inc., Camarillo, CA). A biotinylated secondary antibody (Dako, Botany, NSW, Australia) was then applied followed by streptavidin conjugated to HRP (Dako). The chromogen used was Dako Liquid diaminobenzene (DAB). Twenty high-power (×200) fields were randomly selected for each renal section, and the value of ED-1 positive cells was counted.

The method for terminal deoxynucleotidyl transferase-mediated nick-end labeling method (TUNEL) was performed according to the method as described previously [3]. Briefly, 5-µm thick sections of the kidney were prepared, deparaffinized, and stained by the TUNEL-ABC method. Twenty high-power (×400) fields of the cortex were randomly selected in each section, and the number of apoptotic cells was counted in the renal tubules. The value of apoptotic cells/(apoptotic cells and methyl green stained cells in the renal tubules) was calculated in high-power (×400) fields.

### Fibrotic Scoring and Hydroxyproline Content

Paraffin sections (5 µm) in the kidney sections were stained with Masson’s trichrome. The degree of interstitial collagen deposition was graded as described before [30]. Masson’s trichrome-stained sections were graded (0, no staining; 1, <25% staining; 2, 25 to 50% staining; 3, 50 to 75% staining; 4, 75 to 100% staining of the section). These examinations evaluated the areas overlying the tubular basement membrane and interstitial space while avoiding glomeruli and large vessels. Twenty cortical tubulointerstitial fields that were randomly selected at ×400 magnification were assessed in each rat, and the average for each group was then analyzed.

Hydroxyproline concentration determination for total collagen content was performed in kidney lysates as described from Kivirikko et al. [31]. Briefly, tissue was lysed in 1% Triton, 4 mM EDTA, 1% protease inhibitor cocktail II (Sigma Chemicals) and homogenized with a prechilled mortar and pestle. After centrifugation (10000 rpm at 4°C) both the supernatant and pellet fractions were hydrolysed in 6 M HCl (110°C, 6 h). The data were expressed as µg of collagen per mg total lysate protein using the Bio-Rad Protein Assay (Bio-Rad, Veenendaal, Netherlands).

### Translocation of Nuclear Nrf-2, Mitochondrial Bax and Cytochrome c

We evaluated Nrf-2, I-Nrf-2 (Keap1) in the nuclear and cytosolic proteins. The isolated cortexes were placed in ice-cold isolation buffer containing 0.5 M sacarose, 10 mM Tris-HCl, 1.5 mM MgCl_2_, 10 mM KCl, 10% glycerol, 1 mM EDTA, 1 mM DTT, 2 µg/mL aprotinin, 4 µg/mL leupeptin, 2 µg/mL chymostatin, 2 µg/mL pepstatin, and 100 µg/mL 4-(2 aminoethyl)-benzenesulfonyl fluoride at pH 7.4 and were homogenized by using a tissue grinder [32]. The homogenate was centrifuged at 4,000×*g* for 5 min at 4°C to remove incompletely homogenized fragments and nuclei. The pellet was resuspended in lysis buffer and centrifuged at 12,000×*g* for 20 min at 4°C. Then, the supernatant was resuspended in isolation buffer and the aliquots (nuclear fractions) were stored at −70°C. We used β-actin and LaminA/C Western blot to normalize the cytosol and nuclear fraction protein amount. Hsp70 and β-actin were purchased from Sigma-Aldrich; Nrf-2, I-Nrf-2 (Keap1), and LaminA/C from Santa Cruz Biotechnology (Santa Cruz Biotechnology, Inc., Santa Cruz, CA).

We further investigated the degree of nuclear Nrf-2 activation in this study. Nuclear fractions from fresh kidney tissues were obtained by using nuclear extract kit (Kit 40010, Active Motif, Inc., Carlsbad, CA) and 2.5 µg nuclear extract was used to determine nuclear Nrf-2 activation with a TransAM™ Nrf-2 transcription factor assay (Kit 50296, Active Motif, Inc., Carlsbad, CA). The procedure for measurement of nuclear Nrf-2 activation indicated by the degree of DNA binding of Nrf-2 to antioxidant-response elements was described previously [33].

Cytosolic Bax translocation to mitochondria and mitochondrial leakage of Cytochrome C to cytosol are required for triggering apoptotic pathway [34]. The kidneys were fresh prepared and subjected to differential centrifugation to obtain the mitochondrial and cytosolic protein fractions. Ten µg of protein was electrophoresed using a polyclonal rabbit antihuman Cytochrome C, heat shock protein 60 (HSP60) goat polyclonal antibody (Santa Cruz Biotechology, Inc.) or Bax (Chemicon, Temecula, CA) at 1∶1000.

### Apoptosis, Autophagy and Pyroptosis Proteins Expression by Western Blotting

The expression levels of apoptosis-related proteins including Bcl-2, Bax, and caspase 3 (CPP32), PARP, autophagy-related proteins Beclin-1, Atg5-Atg12 and LC3-II and pyroptosis-related proteins caspase 1 and IL1-β were analyzed by Western blotting in kidney tissues from rats with or without UUO injury. The Western blotting method has been described previously [3], [34]. In brief, the kidney samples were homogenized with a prechilled mortar and pestle in extraction buffer, which consisted of 10 mM Tris-HCl (pH 7.6), 140 mM NaCl, 1 mM PMSF, 1% NP-40, 0.5% deoxycholate, 2% β-mercaptoethanol, 10 µg/ml pepstatin A, and 10 µg/ml aprotinin. The mixtures were homogenized completely by vortexing and kept at 4°C for 30 min. The homogenate was centrifuged at 12,000×g for 12 min at 4°C, the supernatant was collected, and the protein concentrations were determined by BioRad Protein Assay (BioRad Laboratories, Hercules, CA, USA). Antibodies raised against Atg5-Atg12 (Gene Tex, Alton Parkway, Irvine, CA), Beclin-1 (Cell Signaling Technology, Inc., Danvers, MA), caspase 3 (Cell Signaling Technology, Inc.), LC3-II (Cell Signaling Technology, Inc.), caspase 1 (Gene Tex, Alton Parkway, Irvine, CA), IL-1β (Santa Cruz, CA), (Chemicon International Inc., Temecula, CA), Bax (Chemicon), Bcl-2 (Transduction, Bluegrass-Lexington, KY), the activation fragments of caspase 3 (Upstate Biotechnology, Lake Placid, NY), PARP (Cell Signaling Technology, Inc.) and β-actin (Sigma, Saint Louis, MI) were used. SDS-PAGE performed on 12.5% separation gels in the absence of urea was stained with Coomassie brilliant blue. Each lane containing 30 µg of total protein was transferred to nitrocellulose filters. The immunoreactive bands were detected by incubation with the antibody described above, followed by secondary antibody-alkaline phosphatase, and finally with NBT and 5-bromo-4chloro-3-indolyl phosphate, toluidine salt (Roche Diagnostic GmbH, Mannheim, Germany) stock solution for 30 min at room temperature. The density of the band with the appropriate molecular mass was determined semi-quantitatively by densitometry using an image analyzing system (Alpha Innotech, San Leandro, CA, USA).

### Statistical Analysis

The software of SigmaPlot 12.0 (Systat Software, Inc., Chicago, IL, USA) was adapted for graphing and statistical analysis. All values were expressed as mean ± standard error mean (SEM) from 3–10 separate experiments. The differences in all the parameters were compared between control and obstructed kidneys within groups by using Student’s paired *t*-test. One-way analysis of variance was used for establishing differences among groups. Intergroup comparisons were made by Duncan’s multiple-range test. Differences were regarded as significant if *P*<0.05 was attained.

## Results

### UUO Reduced Renal Cortical Blood Flow and O_2_ Tension and Increased Renal ROS

**Figure 1:**
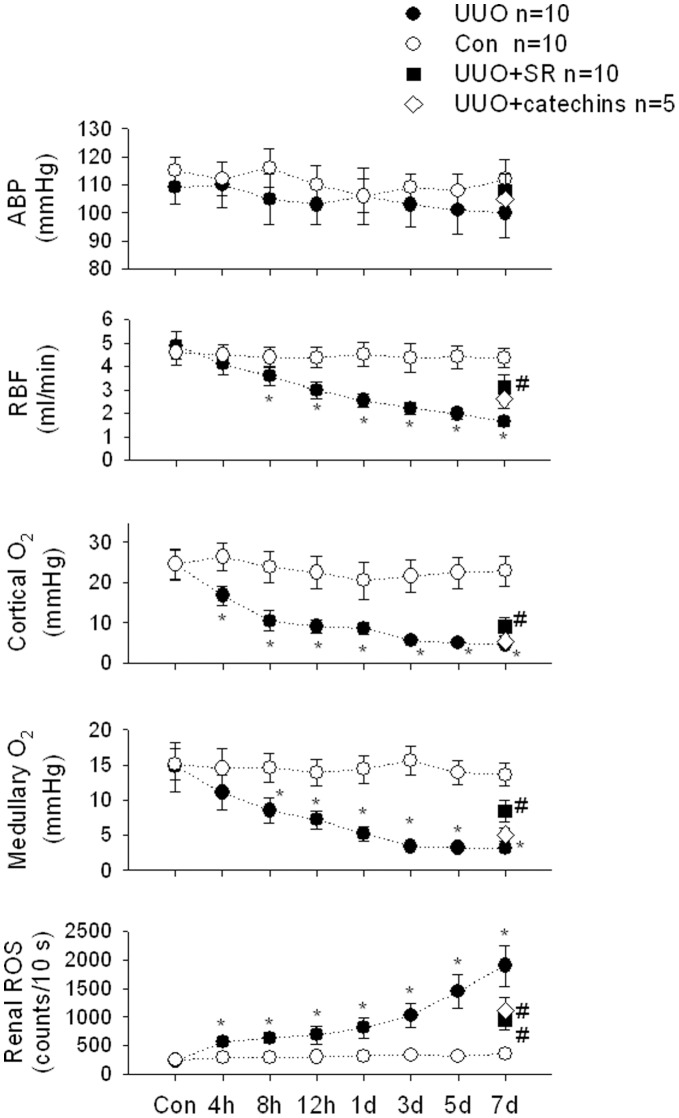
UUO effects on arterial blood pressure (ABP), renal blood flow (RBF), cortical O_2_ and medullary O_2_ tension and renal ROS in kidneys of Control (Con) and UUO groups. UUO significantly reduced RBF and cortical O_2_ and medullary O_2_ tension in a time-dependent manner. An increase in renal ROS is demonstrated in UUO kidneys. SR and catechins treatment significantly improved RBF, cortical and medullary O_2_ tension and decreased renal ROS in the UUO kidneys. Data are expressed as mean ± SEM. *, *P*<0.05 vs. Con group.

We simultaneously measured blood pressure, renal cortical blood flow, O_2_ tension and renal ROS in response to the UUO injury. At 4hUUO, renal blood flow and cortical O_2_ tension are significantly reduced, whereas medullary O_2_ tension is significantly decreased at 8hUUO. Renal ROS are significantly increased at 4hUUO. All the responses are time-dependently affected by the UUO treatment (**Figure 1**). There is no significant change in the blood pressure in response to UUO. The use of SR or catechins displayed a tendency in preserving renal blood flow, cortical and medullary O_2_ tension and decreasing renal ROS amount. It seems that SR confers higher protection than catechins in ameliorating UUO-induced renal dysfunction.

### UUO Induced Renal Fibrosis

**Figure 2:**
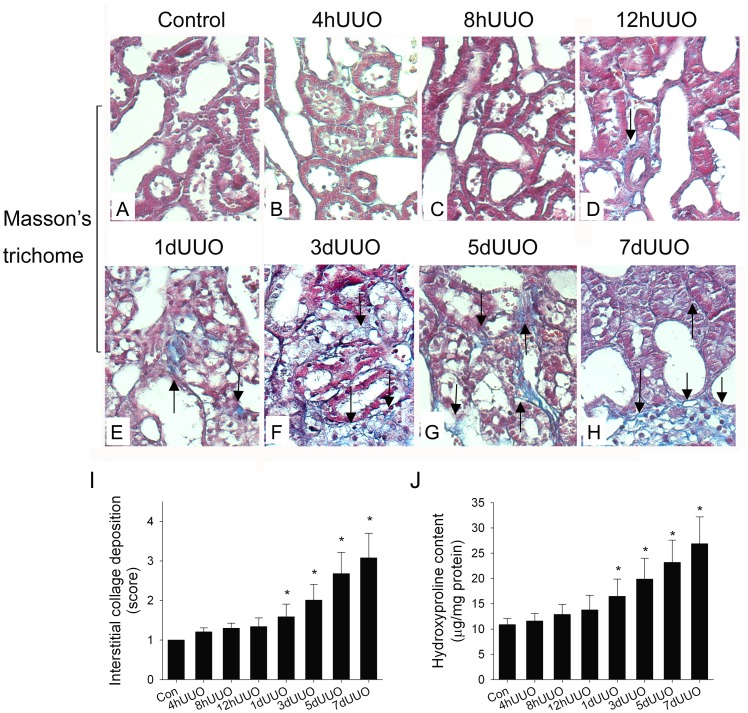
The changes of interstitial collagen deposition and hydroxyproline content in the UUO kidneys. With Masson’s trichrome staining, there is no interstitial collagen deposition in the control kidney (A). Increased Masson stains indicated by blue stain and arrows gradually appear from 4 hUUO (B) and the stains are time-dependently elevated at 8 hUUO (C), 12 hUUO (D), 1dUUO (E), 3dUUO (F), 5dUUO (G) and 7dUUO (H) from representative kidneys. The statistic data of percentage of Masson stains accumulation in the UUO kidneys (n = 10 in each group) is indicated demonstrated in I. The quantitative data of hydroxyproline content (n = 10 in each group) is implicated in J. Significant fibrotic changes are found at 1dUUO-7dUUO. *, *P*<0.05 when compared to the Control (Con) group.

We evaluated UUO-induced renal fibrosis by Masson stain and hydroxyproline content. **Figure 2A** shows that by Masson’s trichrome staining, there is no interstitial collagen deposition (blue stain) in the control kidney. The appearance of Masson stain in the kidney section starts from 4 hUUO (**Figure 2B**) and the blue stains are time-dependently increased at 8hUUO (**Figure 2C**), 12 hUUO (**Figure 2D**), 1dUUO (**Figure 2E**), 3dUUO (**Figure 2F**), 5dUUO (**Figure 2G**) and 7dUUO (**Figure 2H**). Our data show that significant increases in percentage of Masson stains accumulation (**Figure 2I**) and hydroxyproline content (**Figure 2J**) are found after 1dUUO induction.

### UUO Enhanced Autophagy Protein Expression and Stain in the Kidney

**Figure 3:**
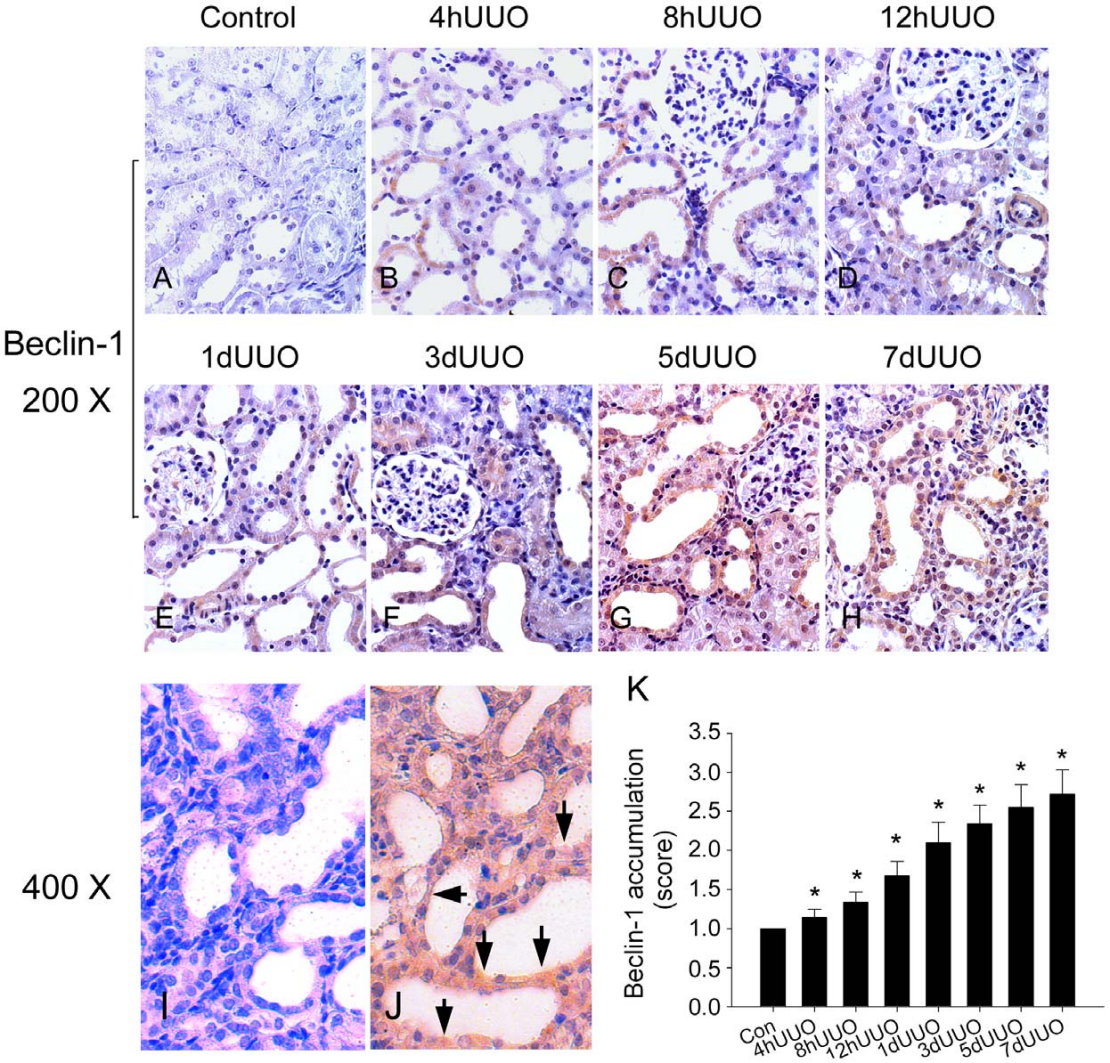
The representative Beclin-1 expression in rat kidneys subjected to different time courses of UUO injury (A–J). All the immunohistochemic graphs are obtained from different animals. There were no Beclin-1 stains in the control kidney (A). Beclin-1 stains appear from 4 hUUO (B) and the stains are time-dependently elevated at 8hUUO (C), 12 hUUO (D), 1dUUO (E), 3dUUO (F), 5dUUO (G) and 7dUUO (H). The statistic data of percentage of Beclin-1 stains accumulation in the UUO kidneys (n = 10 in each group) is demonstrated in K. *, *P*<0.05 when compared to the Control (Con) group.

**Figure 3A** shows that there is no Beclin-1 stains (brown color in cytoplasm) in the control kidney. The appearance of Beclin-1 stains starts from 4 hUUO (**Figure 3B**) and the brown stains are time-dependently increased at 8hUUO (**Figure 3C**), 12hUUO (**Figure 3D**), 1dUUO (**Figure 3E**), 3dUUO (**Figure 3F**), 5dUUO (**Figure 3G**) and 7dUUO (**Figure 3H**). The amplified diagram ×400 clearly shows the Beclin-1 stain in the cytoplasm of renal tubules after 7dUUO (**Figure 3J**) when compared to control tubule (**Figure 3I**). A significant increase in Beclin stains is found after 1dUUO induction (**Figure 3K**).

**Figure 4:**
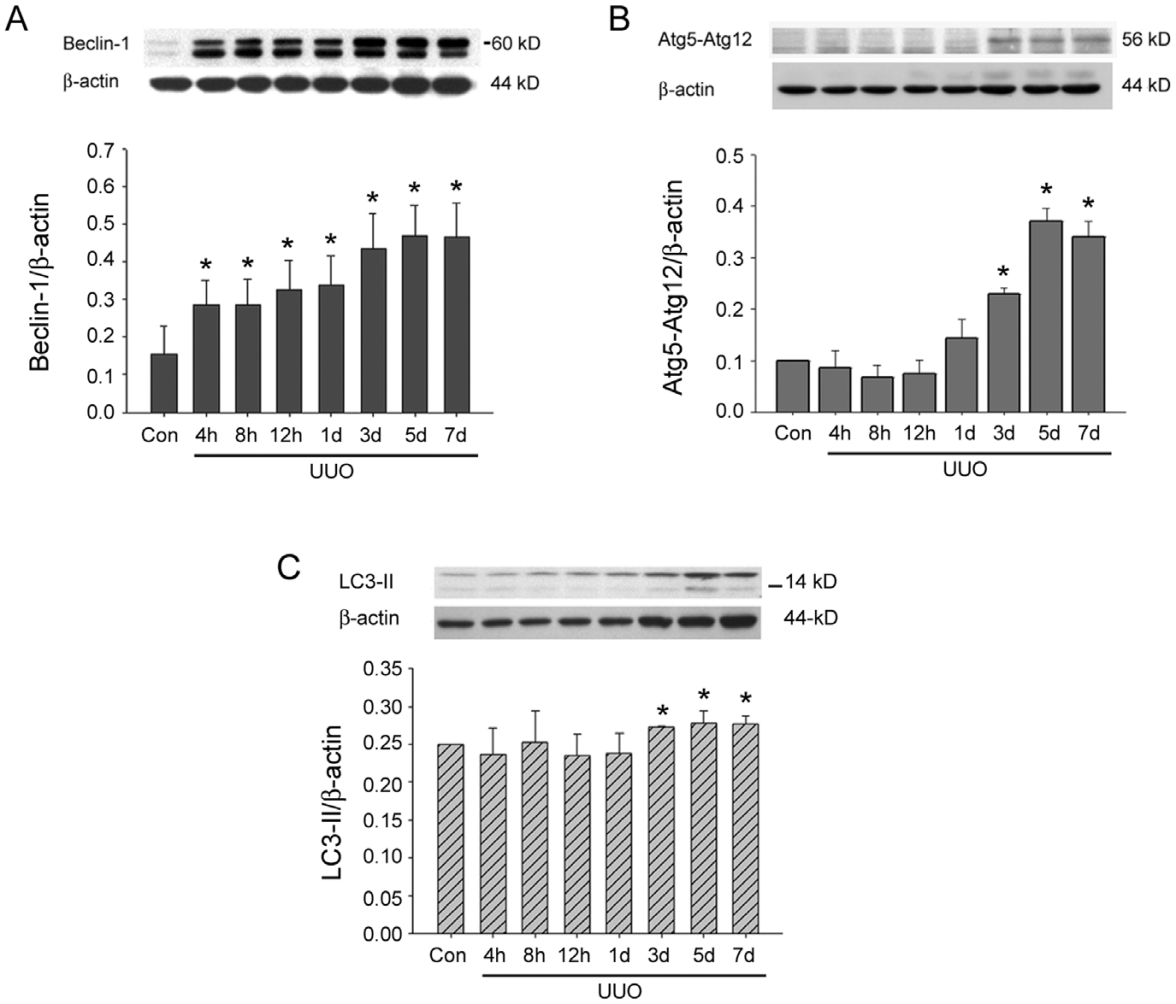
Three autophagy-related proteins expressions in rat kidneys subjected to different time courses of UUO injury. A: Enhanced Beclin-1 protein expression significantly started from 4 h of UUO and further elevated after 3d of UUO. B: Atg5-Atg12 complex significantly enhanced from 3d of UUO and continuously maintained to 7d of UUO. C: LC3-II significantly upregulated from 3d of UUO and maintained to 7d of UUO. All the experiments are performed three times. *, *P*<0.05 when compared to Control (Con) group.

Three autophagy-related proteins expressions in response to UUO injury are demonstrated in **Figure 4**. Enhanced Beclin-1 protein expression significantly appears at 4 hUUO and further elevated after 3dUUO (**Figure 4A**). Atg5-Atg12 (**Figure 4B**) and LC3-II expressions (**Figure 4C**) are significantly activated at 3dUUO and highly expressed until 7dUUO when compared to control group.

### UUO Enhanced Apoptotic and Pyroptotic Protein Expression in the Kidney

**Figure 5:**
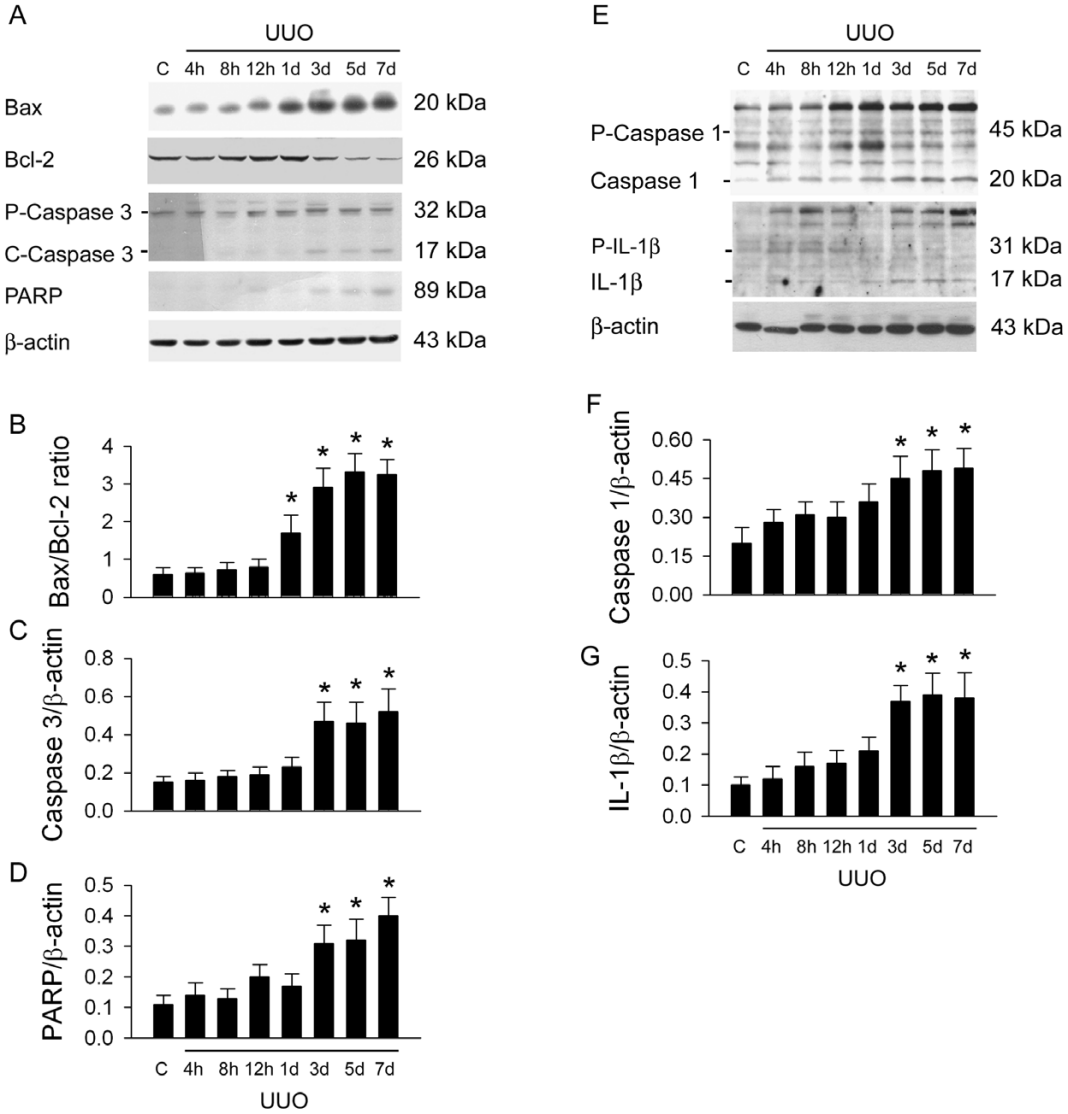
UUO induced apoptosis and pyroptosis related proteins expression in damaged kidneys. Original western blot of apoptosis-related proteins is demonstrated in A. The significant increases in renal Bax/Bcl-2 ratio after 1d UUO (B) and cleaved caspase 3 (C) and PARP expression (D) after 3d of UUO are found. Original tracings of pro- and cleaved caspase 1 and IL-1beta expression are indicated in E. The significant increase in cleaved caspase 1 and IL-1beta expression is noted after 3d of UUO. The experiments are performed three times. *, *P*<0.05 vs. Control (C) group.

We investigated UUO-induced apoptosis and pyroptosis related proteins expression in damaged kidneys **(** **Figure 5** **)**. The western blot of apoptosis-related proteins is demonstrated in **Figure 5A**. The significant increases in renal Bax/Bcl-2 ratio after 1dUUO (**Figure 5B**) and caspase 3 fragments (**Figure 5C**) and PARP expression (**Figure 5D**) after 3dUUO are indicated. Original tracings of pro- and cleaved caspase 1 and IL-1β expression for pyroptosis are indicated in the control and UUO groups **(** **Figure 5E** **)**. The significant increases in cleaved caspase 1 **(** **Figure 5F** **)** and IL-1β expression (**Figure 5G** **)** are recognized after 3dUUO when compared to control group.

### SR Effect on Nrf-2 Translocation, HSP70 Expression, Bax and Cytochrome c Translocation, PARP, Beclin-1, Caspase 1 and IL-1β Expression in the UUO Kidney

**Figure 6:**
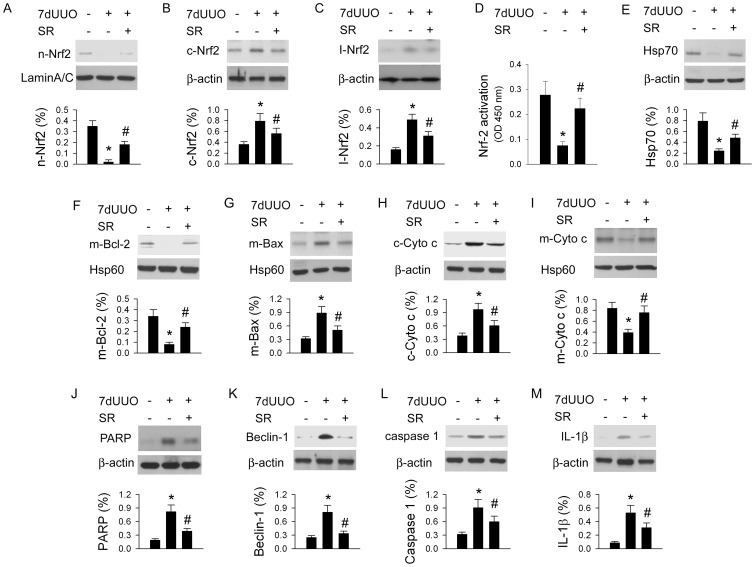
The sulforaphane (SR) effect on Nrf-2-related proteins (A–C), Nrf-2 activation (D), Hsp70 (E), mitochondrial Bcl-2 (F), Bax (G) and cytosolic Cytochrome c (c-Cyto c, H) and mitochondrial Cyto c (m-Cyto c, I) expressions, PARP-apoptosis (J), Beclin-1-autophagy (K), and caspase 1- (L) and IL-1 -mediated pyroptosis (M) in kidneys post 7 d of UUO (7dUUO) injury. 7dUUO decreases nuclear Nrf-2 (n-Nrf-2, A) and increases cytosolic Nrf-2 (c-Nrf-2, B) and inhibitory Nrf-2 (I-Nrf-2, C) levels. 7dUUO significantly depressed Nrf-2 activation (D). SA treatment increases n-Nrf-2 and Nrf-2 activation and decreases c-Nrf-2 and I-Nrf-2 expressions. 7dUUO decreases Hsp70, m-Bcl-2 and m-Cyto c level and increases m-Bax and c-Cyto c levels. SR treatment preserves 7dUUO-induced downregulation in Hsp70, m-Bcl-2 and m-Cyto c levels. 7dUUO enhances renal PARP, Beclin-1, caspase 1 and IL-1β expression. SR administration significantly reduces renal PARP, Beclin-1, caspase 1 and IL-1β expression. All the experiments are performed three times from different rats. *, *P*<0.05 vs. Control group without 7dUUO and SR treatment. #, *P*<0.05 vs. 7dUUO group.

Our data demonstrated that 7dUUO decreases nuclear Nrf-2 (from 0.35±0.5% of control to 0.02±0.02% in 7dUUO, **Figure 6A**) and increases cytosolic Nrf-2 (from 0.36±0.5% of control to 0.79±0.14% in 7dUUO, **Figure 6B**) and inhibitory Nrf-2 (from 0.16±0.02% of control to 0.49±0.06% in 7dUUO, **Figure 6C**) levels in the damaged kidneys compared to control kidneys. 7dUUO inhibited the degree of nuclear Nrf-2 activation (from 0.28±0.05 OD450 nm of control to 0.07±0.02 OD450 nm in 7dUUO, **Figure 6D**). 7dUUO decreases Hsp70 (from 0.78±0.15% of control to 0.24±0.04% in 7dUUO, **Figure 6E**), m-Bcl-2 (from 0.34±0.06% of control to 0.08±0.02% in 7dUUO, **Figure 6F**) and m-Cyto c (from 0.84±0.11% of control to 0.39±0.06% in 7dUUO, **Figure 6H**) level and increases m-Bax (from 0.32±0.04% of control to 0.89±0.14% in 7dUUO, **Figure 6G**) and c-Cyto c (from 0.38±0.06% of control to 0.97±0.14% in 7dUUO, **Figure 6I**) levels. We also found that 7dUUO enhances renal PARP (from 0.19±0.03% of control to 0.82±0.15% in 7dUUO, **Figure 6J**), Beclin-1 (from 0.25±0.04% of control to 0.81±0.17% in 7dUUO, **Figure 6K**), caspase 1 (from 0.32±0.06% of control to 0.91±0.18% in 7dUUO, **Figure 6L**) and IL-1β (from 0.089±0.20% of control to 0.53±0.11% in 7dUUO, **Figure 6M**) expression. SR administration significantly (*P*<0.05) increases nuclear Nrf-2 expression and activation and decreases cytosolic Nrf-2 and inhibitory Nrf-2 expressions. SR treatment also significantly (*P*<0.05) preserves 7dUUO-induced downregulation in Hsp70, m-Bcl-2 and m-Cyto c levels. SR administration significantly (*P*<0.05) reduces UUO-enhanced renal PARP, Beclin-1, caspase 1 and IL-1β expression.

### SR Effect on ED-1, 3-NT, Beclin-1, TUNEL, Caspase 1, IL-1β and TGF-β Expression in UUO Kidney

**Figure 7:**
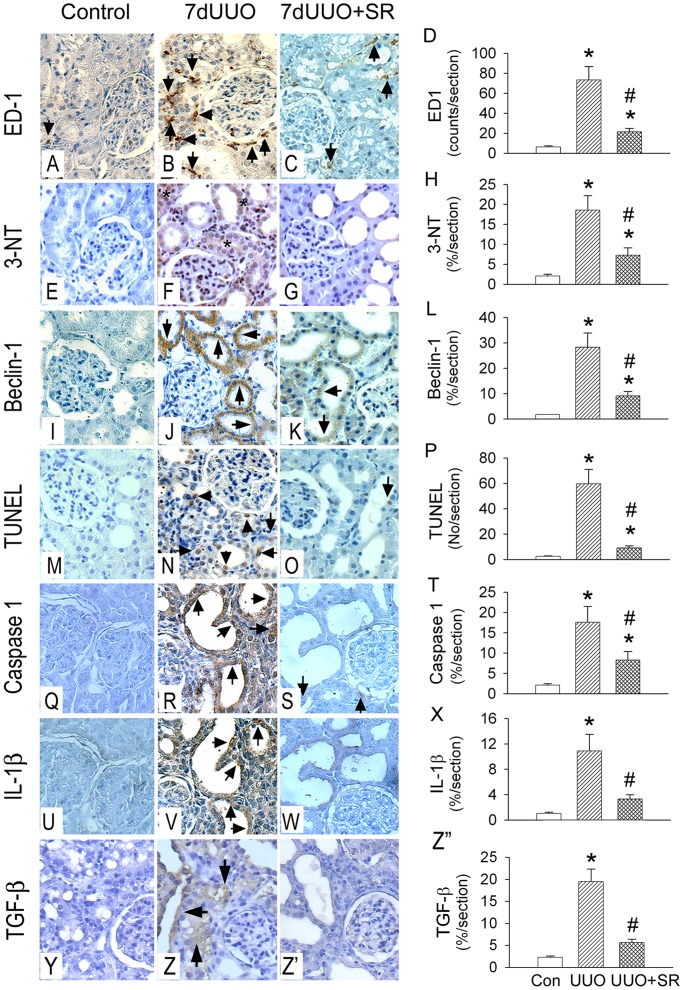
The sulforaphane (SR) effect on inflammation, oxidative stress, autophagy, apoptosis and pyroptosis in the representative kidneys post 7 d of UUO (7dUUO) injury. A–D: ED-1 expression. E–H: 3-NT expression. I–L: Beclin-1 expression. M–P: apoptosis expression by TUNEL stain. Q–T: caspase 1 expression. U–X: IL-1β expresssion. Y–Z”: TGF-β expression. 7dUUO increased ED-1 (B), 3-NT (F), Beclin-1 (J), TUNEL (N), caspase 1 (R), IL-1β (V) and TGF-β (Z) expression when compared to respective control. SR treatment significantly reduced 7dUUO-enhanced ED-1 (C,D), 3-NT (G,H), Beclin-1 (K,L), TUNEL (O,P), caspase 1 (S,T), IL-1 β (W,X) and TGF-β (Z’,Z”) expression (n = 5 in each group). The representative immunohistochemistry is obtained from the serial section of the same rat. *, *P*<0.05 vs. Control (Con) group. #, *P*<0.05 vs. 7dUUO group.

We used immunocytochemistry to localize and examine the oxidative stress, inflammation and three types of cell death in the 7dUUO kidneys with or without SR treatment. 7dUUO significantly (*P*<0.05) increases the number of ED-1 (73.3±13.4 counts/×200 field in 7dUUO vs. 6.3±1.1 counts/×200 field in control, **Figure 7A–D**), 3-NT accumulation (18.6±3.6%/×200 field in 7dUUO vs. 2.1±0.4%/×200 field in control, **Figure 7E–H**), Beclin-1 (28.3±5.6%/section in 7dUUO vs. 1.8±0.2%/section in control, **Figure 7I–L**), TUNEL-positive cells (59.8±11.2 counts/×200 field in 7dUUO vs. 2.5±0.3 counts/×200 field in control, **Figure 7M–P**), caspase 1 (17.6±3.9%/section in 7dUUO vs 2.18±0.24%/section in control, **Figure 7Q–T**), IL-1β (10.9±2.5%/section in 7dUUO vs 1.03±0.21%/section in control, **Figure 7U–X**) and TGF-β (19.5±2.8%/section in 7dUUO vs 2.30±0.31%/section in control, **Figure 7Y–Z** **’’**). SR treatment to the UUO kidneys significantly (*P*<0.05) depressed ED-1 number (21.4±3.5 counts/section), 3-NT accumulation (7.3±1.8%/section), Beclin-1 (9.1±1.7%/section), TUNEL-positive cells (8.9±1.5 counts/×200 field), caspase 1 (8.3±2.1%/section), IL-1β (3.3±0.5%/section) and TGF-β (5.6±0.8%/section) when compared to 7dUUO kidneys.

## Discussion

According to our data and previous studies^1–3,24^, UUO is characterized with a gradually decreased renal blood flow and cortical and medullary oxygen tension and subsequently enhanced renal ROS in a time-dependent manner (**Figure 1**). These led to the increases in tubulointerstitial inflammation (ED-1 infiltration), fibrosis, autophagy, apoptosis and pyroptosis in the damaged kidneys. UUO decreases nuclear Nrf-2 translocation and activity, increases mitochondrial Bax translocation, decreases mitochondrial Bcl-2 expression, increases cytosolic Cytochrome c release, decreases Hsp70 expression and subsequently lead to the autophagy-related protein Beclin-1/Atg5-Atg12/LC3-II expression, apoptosis-related protein Bax/Bcl-2/caspase 3/PARP signaling and pyroptosis-related protein caspase 1 and IL-1β expression in the kidneys. The use of catechins or SR, an Nrf-2 activator, treatment significantly reduces oxidative stress, inflammation, fibrosis and three types of programmed cell death, apoptosis, autophagy and pyroptosis and improves the reduction of renal blood flow in the UUO kidneys.

Metcalfe et al. [35] reported that male rats via testosterone enhanced more proinflammatory TNF-α production and proapoptotic and profibrotic signaling than female rats during UUO, resulting in further increased renal tubular apoptotic cell death, tubulointerstitial fibrosis and renal dysfunction. The occurrence in increased oxidative stress, inflammation, apoptotic cell death, tubulointerstitial fibrosis and renal dysfunction, however, is also found in the UUO kidneys of neonatal and adult female rats [35], [36]. The information indicates in addition to sex hormones, other mechanisms may affect UUO-induced renal dysfunction. Therefore, we used female rats to explore the possible pathophysiologic mechanisms in this study. On the other hand, the gender difference may affect the dosage of Nrf-2 activator, SR, in providing renal protection. Although no gender-specific responses to SR were found in the Sprague-Dawley and Fischer CDF male and female rats [28], the gender differences on SR-induced renoprotective effect between male and female Wistar rats needs to be determined.

Our data showed that hypoxic injury initiated by UUO reduces cortical and medullary O_2_ tension and renal blood flow subsequently resulting in increased oxidative stress. The increased renal ROS *in vivo* by a sensitive MCLA chemiluminescence amplification was appeared earlier around 1hUUO-4hUUO, and the elevated ROS amount was time-dependently enhanced with the increased time of UUO. The intensive intrarenal oxidative stress evoked in UUO kidney was originated from activated macrophages or damaged tubular-derived ROS production, because we clearly recognized increased ED-1 (macrophages) infiltration and oxidative stress, 3-NT stains, located primarily in the tubulointerstitial area of the UUO kidney. We have also confirmed our data with previous findings by Yeh et al. [2] that increased renal ROS occurred at the early period of 1hUUO, enhanced ER stress marker ERS25, ER molecular stress chaperone GRP78 and p-JNK and caspase 12 protein expression at 4hUUO and resulted in apoptosis formation around 8hUUO-1dUUO. We further found that after enhanced ROS, ER stress and apoptosis formation, the autophagic and pyroptotic cell death examined by western blotting were identified at 3dUUO stage. The appearance of the autophagy marker for Beclin-1 at initiation stage, Atg5-Atg12 at elongation stage and LC3-II at maturation stage was 4h, 3d and 3d after UUO injury, respectively. Pyroptosis indicated by caspase 1/IL-β stain and western blot was recognized at 3dUUO. Based on these information, we suggest that UUO-evoked ROS comes before the activation of ER stress (4 hUUO), apoptosis (8h-1dUUO) [2], LC3-II-autophagic cell death (3dUUO) and caspase 1/IL-1β pyroptosis (3dUUO). Excess ROS production from damaged tubular cells or infiltrated leukocytes like ED-1-macrophages leads to tubular apoptosis and autophagy [10], [18] and contributes to the pathogenesis of tubulointerstitial fibrosis [37], chronic inflammation and caspase 1-mediated IL-1β expression (recognized by pyroptosis in the present study) in the obstructed kidney. All these results imply that UUO impairs renal function via the mechanisms of oxidative stress, inflammation, fibrosis [2], [38], apoptosis, autophagy and pyroptosis (in the present study).

A mitochondrial dysfunction indicated by the increased mitochondrial Bax translocation and cytosolic Cytochrome c release was noted after UUO injury. The impaired mitochondria via decreasing blood and oxygen supply could induce more ROS production from the impaired electron transport system and several caspase/enzyme activities to trigger Bax/Bcl-2/caspase 3/PARP signaling for apoptosis, Beclin-1/Atg5-Atg12/LC3-II signaling for autophagy and caspase 1/IL-1β signaling for pyroptosis production in the UUO kidneys. Exacerbated ROS facilitates autophagic cell death or type II programmed cell death associated with the increased apoptotic cell death [10]. Bax and Bcl-2 can regulate apoptosis, autophagy and pyroptosis formation [9]. Overexpression of Bcl-2 or Bcl-xL by adenoviral *bcl-2*/*bcl-xL* transfection or in *bcl-2* transgenic mice inhibited ROS-induced tubular apoptosis and autophagy [10], [18], [34]. Furthermore, Bcl-2 and Bcl-xL bind and suppress NLR-family protein NALP1, a nucleotide-dependent activator of cytokine-processing protease caspase-1, to inhibit caspase-1 activation and IL-1β production [19]. Bcl-2-deficient macrophages exhibit more caspase-1 processing and IL-1β production, whereas Bcl-2-overexpressing macrophages implicate less caspase-1 processing and IL-1β production [19]. These findings imply an important role of Bcl-2 family in the regulation of three types of programmed cell death.

Bcl-2 has a dual subcellular localization in mitochondria of renal tubules [34], [39]. Bcl-2 disrupts the proapoptotic proteins of Bax and acts as channel proteins in the mitochondrial membrane to prevent the mitochondrial release of Cytochrome c for induction of caspase cascade and apoptosis [17], [34]. Increased cytosolic Bax translocation to mitochondria triggers cytosolic Cytochrome c release for inducing apoptosis, whereas increased Bcl-2 and/or Bcl-xL inhibits cytosolic Cytochrome c release for depressing apoptosis [15]–[17]. In the proximal tubular cells, increased ROS by ischemia/reperfusion down-regulates Bcl-2 or up-regulates Bax to alter mitochondrial membrane permeability, to trigger mitochondrial Cytochrome c release to cytosol and to activate caspase 3 expression [34]. Enhancement of Bcl-2 expression by alpha-MSH treatment prevented the Bax and TGF-β protein increase and induced Bcl-2 protein increase, together with reduction of apoptosis, inflammation, and tubulointerstitial fibrosis [40]. Bcl-2 augmentation by adenoviral *bcl-2* transfer or transgenic *bcl-2* protects renal tubular epithelial cells from ischemia/reperfusion injury by suppressing autophagosomal degradation of injured mitochondria, autophagic cell death and apoptosis [18], [34]. Bcl-2/Bcl-xL suppresses NALP1-inflammasomes-mediated caspase-1 activation and IL-1β production (pyroptosis) [19]. Our data demonstrated that UUO increased Bax/Bcl-2 ratio and caspase 3/PARP expression for apoptotic cell death, enhanced Beclin-1, Atg5-Atg12 and LC3-II for autophagic cell death and promoted caspase 1/IL-1β expression for pyroptotic cell death in the kidney. These data inform that Bcl-2 family participates in three types of programmed cell death.

Nrf-2/Keap1 signaling molecules can affect NF-κB signaling-mediated inflammation and two types of programmed cell death, autophagy and apoptosis [25]. In the present study, we further explored that activating Nrf-2 signaling can inhibit caspase-1/IL-1β/pyroptosis and TGF-β/fibrosis induction. Under hypoxic condition in our UUO model, the increased cytosolic I-Nrf-2 (Keap1) protein level was associated with the decreased nuclear Nrf-2 expression and activity and Hsp70 expression in the damaged kidney. This finding is similar to the report of Rinaldi Tosi et al. [31]. We also found that decreased nuclear Nrf-2 translocation and activity is also accompanied with the mitochondrial dysfunction, which includes the decreased mitochondrial Bcl-2 expression and increased mitochondrial Bax and cytosolic Cytochrome c release in the 7dUUO kidney. The use of Nrf-2 activator, SR, treatment preserved nuclear Nrf-2 expression and mitochondrial function. We think that the increased Nrf-2 expression and activity and the upregulated antioxidant response elements like heme-oxygenase 1 and Hsp70 could protect mitochondrial integrity. Increased oxidative stress from a butylated hydroxyanisole metabolite elevates mitochondrial ROS amount and depresses Nrf-2-mediated antioxidant responses [41]. Upregulating Nrf-2 signaling provides cardioprotection and Nrf-2-knockout loses cardioprotection [27]. Knockdown of Nrf-2 increases autophagic cell death [24], [42]. Genetic ablation of Nrf-2 decreases Nrf-2 binding activity, the expression and activity of antioxidant enzymes and exacerbates the neurologic deficit and inflammation (release of IL-6 and IL-1β) after spinal cord injury in mice [43]. Nrf-2^−/−^ mice endangers higher ROS production, oxidative DNA damage and renal injury than Nrf-2^+/+^ mice in diabetic nephropathy [44]. Mechanistic studies showed that overexpression of Nrf-2 inhibited TGF-β, whereas knockdown of Nrf-2 by siRNA enhanced TGF-β and fibronectin production [44]. Nrf-2 activators administered 2 weeks after diabetes induction significantly attenuated metabolic disorder symptoms associated with diabetes and reduced oxidative damage and the expression of TGF-β and extracellular matrix proteins [13]. Our data informed that Nrf-2 activation by SR reduces TGF-β expression and hydroxyproline level resulting in decreasing UUO-induced fibrosis and mitigated renal injury. These results were consistent with previous findings [44], [45] that antioxidants treatment improved renal function, histology, and vascular permeability in Nrf-2^−/−^ mice with ischemic damage. We have also compared the antioxidant efficiency between SR and catechins and recognized that SR confers higher renal protection than catechins in the improvement of renal oxygen tension and the decrease of ROS amount. However, the beneficial mechanism should be determined in future. The activation of Nrf-2-related defense mechanisms before disease development or during the early period of renal diseases is the critical point for intervention to attenuate ROS-induced three types of programmed cell death and the disease progression. Overall, the present study showed that SR administration via increased nuclear Nrf-2 translocation and activity ameliorated oxidative stress, mitochondrial dysfunction, apoptosis, autophagy, pyroptosis, tubulointerstitial inflammation, fibrosis and renal blood flow in the UUO kidney suggesting a therapeutic potential in future.

In conclusion, we found that diminished nuclear Nrf-2 translocation contributes to oxidative stress and mitochondrial dysfunction in damaged cells of the UUO kidney. UUO-induced inflammation and oxidative stress enhance three types of programmed cell death, autophagy, apoptosis and pyroptosis formation in the damaged kidney through the mitochondrial dysfunction and inhibition of nuclear Nrf-2 translocation. The use of Nrf-2 activator to enhance nuclear Nrf-2 translocation and activity may be applied to renal diseases for preserving mitochondrial function and mitigating oxidative stress, inflammation, fibrosis and three types of programmed cell death.
